# Imprinting of Molecular Recognition Sites on Nanostructures and Its Applications in Chemosensors

**DOI:** 10.3390/s8128291

**Published:** 2008-12-15

**Authors:** Guijian Guan, Bianhua Liu, Zhenyang Wang, Zhongping Zhang

**Affiliations:** Key Laboratory of Biomimetic Sensing & Advanced Robot Technology, Institute of Intelligent Machines, Chinese Academy of Sciences, Hefei, Anhui 230031, P.R. China.

**Keywords:** Molecularly imprinted polymers, Nanostructures, Chemical detection, Sensors

## Abstract

Biological receptors including enzymes, antibodies and active proteins have been widely used as the detection platform in a variety of chemo/biosensors and bioassays. However, the use of artificial host materials in chemical/biological detections has become increasingly attractive, because the synthetic recognition systems such as molecularly imprinted polymers (MIPs) usually have lower costs, higher physical/chemical stability, easier preparation and better engineering possibility than biological receptors. Molecular imprinting is one of the most efficient strategies to offer a synthetic route to artificial recognition systems by a template polymerization technique, and has attracted considerable efforts due to its importance in separation, chemo/biosensors, catalysis and biomedicine. Despite the fact that MIPs have molecular recognition ability similar to that of biological receptors, traditional bulky MIP materials usually exhibit a low binding capacity and slow binding kinetics to the target species. Moreover, the MIP materials lack the signal-output response to analyte binding events when used as recognition elements in chemo/biosensors or bioassays. Recently, various explorations have demonstrated that molecular imprinting nanotechniques may provide a potential solution to these difficulties. Many successful examples of the development of MIP-based sensors have also been reported during the past several decades. This review will begin with a brief introduction to the principle of molecular imprinting nanotechnology, and then mainly summarize various synthesis methodologies and recognition properties of MIP nanomaterials and their applications in MIP-based chemosensors. Finally, the future perspectives and efforts in MIP nanomaterials and MIP-based sensors are given.

## 1. Introduction

All over the world, billions of dollars are spent annually on chemical/biological detections related to medical diagnosis, environmental monitoring, public security and food safety because lab analysis using expensive equipment is usually cumbersome and time-consuming. Therefore, there has been a pressing societal need for the development of chemo/biosensors for the detection of various analytes in solution and atmosphere, which are both less expensive and simpler to construct and operate. Although considerable progress was made in the past several decades, the chemo/biosensor field remains underdeveloped and at a low level of commercialization because of the lack of alternative strategies and multidisciplinary approaches. Only a few chemo/biosensors for simple analytes have been able to meet commercial requirements with detection sensitivity, selectivity, accuracy and reliability approaching that of experimental equipment [1-5]. However, the recent developments of novel chemosensory materials and fabrication technologies may provide many potential opportunities for the development of a new generation of chemo/biosensors [6-11]. Thus, the explorations on chemo/biosensors based on novel sensing concept have attracted growing interest in recent years.

**Figure 1.**
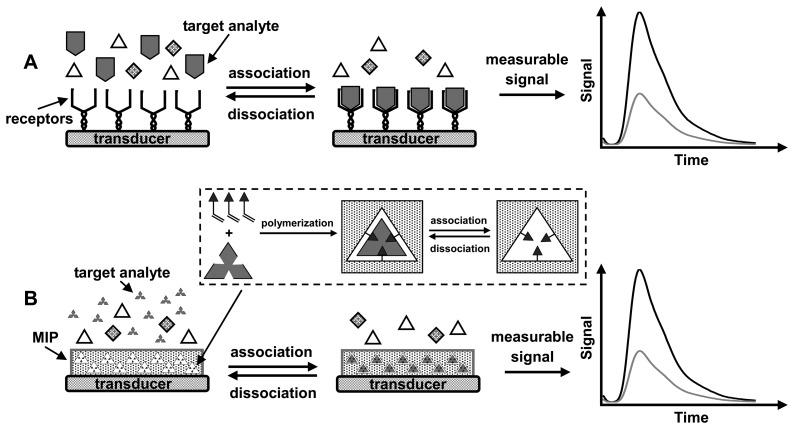
Schematic representations of (A) antibody-based chemosensor and (B) MIP-based biomimetic sensor. Inset in (B) shows the concept of molecular imprinting.

As shown in Figure 1A, a typical chemosensor consists of two main components: the chemosensory materials (receptors) that can selectively binding target analytes and the efficient transducer that can transform the binding events into a readable signal output related to the analyte concentration in the sample [8]. The efficiency of chemosensors is largely dependent on the selectivity and sensitivity of the used sensory materials to a target species. The traditional approaches are to immobilize biological receptors (e.g., enzymes, antibodies, DNA, proteins, etc) on the surface of a physical transducer to provide selective binding of analytes [6, 8-11]. However, the small surface area and non-tunable surface properties of transducers greatly limit the efficiency of chemosensors, especially for the detection of ultratrace analytes. Recently, nanomaterials have found a wide range of applications as a material foundation of chemosensors, and have exhibited various degrees of success in the improvement of detection sensitivity and selectivity [12-16]. Nanomaterials themselves can also form a novel platform of chemical/biological detections due to their unique electrical, optical, catalytic or magnetic properties [12, 13]. Moreover, the large surface-to-volume ratio and good dispersivity of nanomaterials provide a huge adsorptive surface for enriching target species [14-16]. In particular, the chemical immobilization of biological receptors such as antibodies and enzymes or organic ligands at the surface of nanostructures will further enhance the sensitivity and selectivity of detections [12, 17]. Therefore, chemo/biosensors based on functionalized inorganic or organic nanomaterials have widely been explored for the detections of various analytes, such as quantum dots for the fluorescent detections of nitroaromatic explosives [18], DNA [19] and enzyme activity [9, 10], and functionalized carbon nanotubes for the electrochemical detections of various organic/biological analytes [20, 21].

Although biological receptors have specific molecular affinity and have been widely used in diagnostic bioassays and chemo/biosensors, they are often produced via complex protocols with a high cost and require specific handling conditions because of their poor stability, and the natural receptors for many detected analytes don't exist [22-25]. Thus, there has been a strong driving force in synthesizing artificial recognition receptors. Molecular imprinting is one of the most efficient strategies that offer a synthetic route to artificial recognition systems by a template polymerization technique [23-41]. In molecular imprinting technology, the specific recognition sites can be spatially organized by imprinting template molecules in a polymeric organic/inorganic matrix through the template-monomer complexes such as covalent and non-covalent interactions, as drawn in the inset of Figure 1B. After crosslinking copolymerization, the removal of templates from the crosslinked matrix generates the recognition cavities complementary to the shape, size and functionality of templates, leaving the steric and chemical information of the imprinted molecules. Therefore, the target species can selectively rebind into the molecularly imprinted polymers (MIPs) through the specific interaction with these imprinted sites. The synthesis technique is simple and cheap, and the resultant MIP materials exhibit high selectivity, excellent mechanical strength, durability to heat, acid and base conditions and better engineering possibility than biological counterparts [23-25]. Moreover, the introduction of synthetic design into molecular imprinting strategy can even make a host element suitable for the analyte for which the natural receptor does not exist. These characteristics allow MIP materials as recognition elements to be used in a wide range of fields such as life, pharmaceutical and environmental sciences [36-38]. Although the main applications continue to be in selective separation, MIP-based sensors for the detection of active molecules, pharmaceuticals and environmental pollutants are perhaps the most challenging, and have attracted considerable interest in recent years [42-46]. When the combination of MIP materials with a transducer in a suitable format, the sensors with MIP recognition can identify and quantify a target species by converting the analyte-MIP binding event into a physically readable signal, as shown in Figure 1B.

Many papers have reviewed the traditional imprinted materials and their applications [47-51]. However, thus far there have not been any reviews dedicated to the evaluation of molecularly imprinted nanomaterials. Thus, the current review will focus on the recent advance on imprinting of molecular recognition sites at nanostructures and its applications in chemo/biosensors.

## 2. Comparison of Normal MIP Materials with MIP Nanomaterials

### 2.1. Limitations of Traditional Imprinted Polymer Materials

Molecular imprinting can generate recognition cavities with the steric and chemical information of template molecules in a crosslinked polymeric network. However, the effectiveness of molecular imprinting is greatly dependent on the bond nature of template-monomer complex [35-38], the form of imprinted materials [14-16, 52], and the rigidity of polymeric matrix [53-59]. While various imprinted materials were synthesized by different strategies, the imprinted materials ideally suitable for the molecular recognition elements have yet to be explored, because of their small binding capacity and slow binding kinetics. Although the conventional imprinting protocol is simple and effective, there are at least several critical factors to obstruct the applications of molecularly imprinted materials as the artificial receptors in analytical chemistry. Firstly, most of imprinted polymers are highly crosslinked bulky polymers with irregular shape. Although a grinding process is usually used, the extraction of original templates located at the interior area of bulky materials is quite difficult because of the high crosslinking nature of imprinted materials, which reduces the capacity of rebinding target analytes [53-55]. Furthermore, if the generated cavities are not at the surface or in the proximity of the material's surface, the high resistance to mass transfer will still hinder target species from accessing the deep imprinted cavities, thus slowing the kinetics of target analyte binding [56, 57]. Moreover, the rigid polymeric matrix greatly reduces the conformational freedom of molecular recognition by excluding any further chain mobility [58, 59]. Secondly, the uncontrollable random polymerization always suffers from the heterogeneity of the imprinted sites in the formed polymer matrix [60, 61]. One of main reasons is that vinyl functional monomer and divinyl crosslinking agent have essentially different polymerizing abilities, and the used amount of crosslinking agent is much larger than that of functional monomer. Finally, the analyte binding at MIP materials usually lacks signal output due to the poor assembly ability at the surface of transducer, limiting their use in chemical detections or bioassays [43]. Therefore, optimization of the imprinted materials at a molecular level to thus provide a better antibody/enzyme mimic remains a great challenge.

### 2.2. Various Ongoing Explorations on Novel Imprinting Strategies

One of the attempts to address these problems is that the imprinted materials are prepared in an optimizing form that control templates to be situated at the surface or in the proximity of materials surface [62-77]. Mosbach [62] first reported the surface molecular imprinting strategy by covalent immobilization of template molecules at the surface of a solid substrate. After the imprinting polymerization and the removal of substrate, all of templates were situated at the surface of imprinted materials, providing a complete removal of templates and an excellent accessibility to target species, and conformational flexibility of recognition. The approach is well suited for imprinting of proteins [63-65], cells [66, 67] and virus [68] immobilized onto a flat substrate, because the templates with large sizes are more difficultly removed in traditional MIPs. However, the surface area of the substrate is very limited, and accordingly the total number of the resultant recognition sites is always very small. Another similar alternative proposed by Sellergren [72-74] is to prepare the molecularly imprinted films by chemical immobilization of azo-initiators/chain-transfer agents at the surface of substrates, followed by initiating an imprinting polymerization reaction of organic monomers on substrate. Although the immobilization of initiators is often complex and chemically instable, the method should be adaptable to supports with different morphologies. On the other hand, Zimmerman [75, 76] developed a monomolecular dendritic imprinting strategy by polymerization of dendrimer with vinyl end groups. Each polymerizing dendrimer contains only one imprinted site after removal of templates. Recently, the use of natural polymers for preparing molecularly imprinted materials may also provide a potential solution to the above concerns [58, 59]. The types of gel-state materials, such as chitosan hydrogels, are formed with a lower crosslinking density than the traditional imprinted materials synthesized from polymeric monomers, and thus they are more flexible and have a greater conformational freedom of molecular recognition.

### 2.3. Advantages of Molecularly Imprinted Nanomaterials

**Figure 2.**
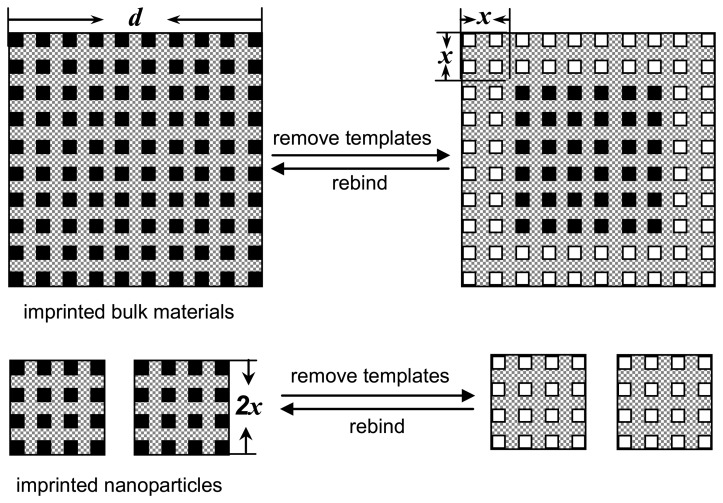
The schematic illustration of the distribution of effective binding sites in the imprinted bulky materials and the nanosized, imprinted particles after the removal of templates is done. Reprinted with permission from [16]. Copyright 2007 American Chemical Society.

Recently, the introduction of novel nanotechnologies and surface chemistry into molecular imprinting strategy has attracted considerable research interest because of the wider applications of imprinted nanomaterials in bioassays and chemosensors [14-16, 78, 79]. In general, the gap between biological and artificial sensors is mostly the usually much higher affinity and thus sensitivity reached with e.g. antibodies. The molecular imprinting nanotechnologies are expected to greatly enhance the molecular affinity of MIP materials, and thus provide a wider range of applications approaching to biological receptors [80]. Nanostructured, imprinted materials have a small dimension with extremely high surface-to-volume ratio, so that most of template molecules are situated at the surface and in the proximity of materials surface. Figure 2 illustrates the distribution of effective binding sites in the imprinted bulky materials and imprinted nanoparticles after the extraction of templates is done [16]. We assume that these templates located within *x*-nanometers from the surface can be removed in the bulky materials with a scale of *d*, and the resultant imprinted sites can be accessed to target species. The effective volume of imprinted materials that can rebind target species is [*d*^3^-(*d*-2*x*)^3^]. In general, the *x* value is very small for highly crosslinked bulky materials although porogens or solvents are usually used in the imprinting process. If the imprinted materials with the same size are prepared in the form of nanostructure with a scale of 2*x* nm, all of templates can be completely removed from the highly crosslinked matrix, and these resultant sites are all effective for the binding of target species. In the case of nanosized particles, most of imprinted sites are situated at the surface or in the proximity of surface. Therefore, the forms of imprinted materials are expected to greatly improve the binding capacity and kinetics and site accessibility of imprinted materials. Compared with the imprinted films and surface-imprinted materials, the imprinted nanomaterials have a higher affinity and sensitivity to target analyte, and a more homogeneous distribution of recognition sites.

On the other hand, the low-dimensional nanostructures with imprinted sites have very regular shapes and sizes, and the tunable flexibility of shapes and sizes. The imprinted nanomaterials have also better dispersibility in analyte solutions and thus greatly reduce the resistance of mass transfer, exhibiting a fast binding kinetics [14, 15]. In particular, novel nanostructure assembly technologies have achieved a wide success in building various nanodevices [12, 13]. The imprinted nanomaterials with well-defined morphologies can feasibly been installed onto the surface of devices in a required form for many applications in nanosensors and molecular detection.

## 3. Imprinting of Molecular Recognition Sites at Nanostructures

Molecular imprinting polymerization can occur usually in a fast and uncontrollable manner due to the use of a large amount of crosslinking agents. Oriented growth, as in the formation of inorganic crystalline nanostructures, almost never occurs in the preparation of imprinted polymers. Therefore, to synthesize MIP nanostructures one needs more external control over the polymerization process, such as the use of inorganic templates [16, 73, 78], extra nanospace confinement [14, 15, 79], chemical microemulsion [56, 57] and self assembly [81]. Furthermore, the external controls must not weaken the interaction between template molecules and functional monomers, and hinder the occurrence of polymerizing reaction. Recently, these novel strategies have widely been explored and led to the formation of various molecularly imprinted nanostructures [82-107].

### 3.1. Nanospheres

Normal bulky MIPs need a grinding process to obtain small particles for many applications. Recently, much attention has been paid on the preparation of MIP nanospheres because of the high yields and relatively simply control over size and distribution [53, 82-85]. The spherical form of MIPs is better suited for the efficient use in chromatography, solid-phase extraction, and other flow-through techniques, and the loading onto a membrane for template extraction. Li and co-workers [82] recently designed a kind of block copolymers useful for synthesizing MIP nanospheres. One block with functional group provides hydrogen-bond interaction with templates and simultaneously allows the formation of micro micelles, and another block containing polymerizable groups plays the role of crosslinking agent, leading to the formation of uniform nanospheres. The measurement results showed that the shape and size of the MIP particles are of critical importance for the imprinting efficiency. With the smaller sizes and better-defined morphologies, the MIP nanospheres achieved higher affinity and selectivity, and better site accessibility. The MIP nanospheres with 100-nm size had a higher binding capacity, which was 2.5 fold that of normal bulky particles with 5-μm size. Ciardelli [85] demonstrated the formation of cholesterol-imprinted nanospheres through a crosslinking polymerization of methacrylic acid in a dilute precursor solution, which is relatively simpler than microemulsion. Typically, Kempe [83] developed a more straightforward route to prepare spherical MIP beads through the formation of droplets of prepolymerization solution directly in mineral oil, followed by transformation of the droplets into solid spherical beads by photoinduced free radical polymerization. The imprinting polymerization was completed within only 10 min under a high efficiency UV lamp. The rebinding capacity for the beads was 1.6 times that for the corresponding ground polymer monoliths. This might be the result of a better accessibility of the recognition sites in the beads with high surface-to-volume ratio.

### 3.2. Core-Shell Imprinted Nanoparticles and Imprinted Nanocapsules

In order to locate the imprinted sites at the surface or at surface proximity of materials, recently many researchers have explored the preparation of core-shell imprinted nanoparticles via the use of inorganic/organic template colloidal particles. After surface chemical modifications, a variety of support cores including silica [16, 73, 78], polystyrene [56, 57], silver [86], chitosan [87], and Fe_3_O_4_ nanoparticles [88, 89] could direct the occurrence of imprinting polymerization at the surface of these template particles, leading to the formation of MIP shells. If the MIP shell is sufficiently thin, the template can be extracted completely, following the creation of the imprinted sites at the polymer shells. Thus, target species can more easily access the imprinted sites. A typical strategy is that silica core particles were first modified with azo-initiators/chain-transfer agents by surface chemistry, followed by initiating an imprinting polymerization reaction, leading to the formation of imprinted shells at the surface of silica particles [73, 78]. On the other hand, core-shell emulsion polymerization has also successfully control the template molecules to be located at the surface of the formed particles [56, 57]. The approach is simpler and more straightforward, and is expected to improve the effects of molecular imprinting. In particular, the resultant magnetic core-shell imprinted particles are very useful in separation and collection of target species [88-90].

**Figure 3.**
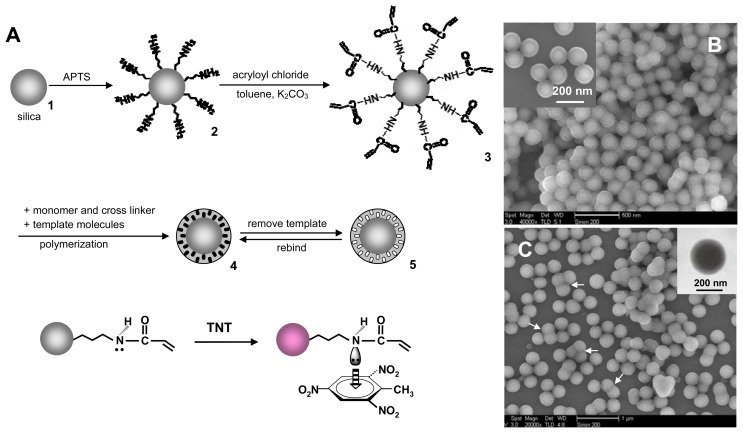
(A) Schematic illustration of molecular imprinting on silica nanoparticles. (B) SEM image of SiO_2_@TNT-MIP particles with 100-nm-sized core (inset is high-magnification SEM image). (C) SEM image of SiO_2_@TNT-MIP particles with 200-nm-sized core (inset is high-magnification TEM image). Reprinted with permission from [16]. Copyright 2007 American Chemical Society.

Recently, our group [16] has developed a surface functional monomer-directing strategy for the synthesis of high-quality SiO_2_@MIP nanoparticles with a high density of molecular recognition sites, as shown in Figure 3. The silica cores were chemically modified with acrylamide monomers (Figure 3A). The vinyl functional monomer layer can not only direct the selective occurrence of imprinting polymerization at the surface of silica through the copolymerization of vinyl end groups with functional monomers, but also drive 2,4,6-trinitrotoluene (TNT) templates into the formed polymer shells through the charge-transfer complexing and hydrogen bond interactions between TNT and the functional monomer layer. The two basic processes led to the formation of uniform core-shell TNT-imprinted nanoparticles (Figures 3B and C) with a controllable shell thickness and a high density of effective recognition sites. The high capacity and fast kinetics to uptake TNT molecules had been demonstrated, and the density of effective imprinted sites in the nanoshells was nearly five times that of traditional bulky particles. A critical value of shell thickness (∼25 nm) for the maximum rebinding capacity was determined by testing the evolution of rebinding capacity with shell thickness, which provides new insights into the effectiveness of molecular imprinting and the form of imprinted materials.

**Figure 4.**
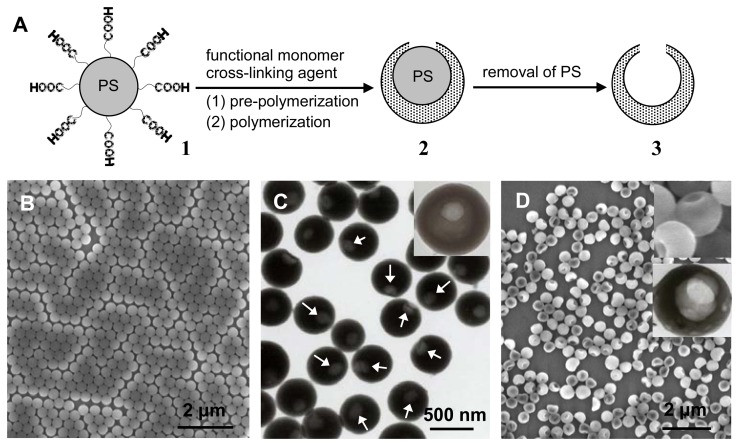
The schematic illustration and an experimental example for preparing MIP nanocapsules with a single hole: (A) Single-hole nanocapsules were synthesized by the consecutive two-step polymerization at the surface of carboxyl-capped polystyrene (PS) beads, followed by the dissolution of PS cores with tetrahydrofuran; (B) SEM image of original carboxyl-capped PS beads; (C) TEM image of PS@Poly(AA-EGDMA) microspheres (inset is a high-magnification TEM image); (D) SEM image of Poly(AA-EGDMA) nanocapsules with a single hole (the upper and bottom insets are high-magnification SEM and TEM images, respectively). Reprinted with permission from [52]. Copyright 2007 Wiley-VCH Verlag GmbH & Co. KGaA.

It is very interesting that we recently prepared the TNT-imprinted nanocapsules with a single hole [52], as shown in Figure 4. The single-hole nanocapsules were synthesized through the direct polymerization and crosslinking reactions occurring at the surface of carboxyl-capped polystyrene colloids. The formation mechanism of a hole is based on the concomitant microphase separation and symmetrical volume shrinkage of shell materials during the imprinting polymerization, providing a reproducible approach for creating holes in the imprinted polymer shells. The TEM image of Figure 4C shows that a single hole was formed at the polymer shell of the core-shell particles, as indicated with arrows. The single-hole nanocapsules were subsequently produced by the dissolution of polystyrene cores with tetrahydrofuran, as shown in Figure 4D. The experimental findings allow the imprinting of organic/biological molecules at the single-hole nanocapsules toward the specific high-capacity uptake of target species. The nanocapsules with two open surfaces provide more complete removal of the templates, better site accessibility and larger surface area. The TNT rebinding capacity of nanocapsules is about 4 fold that of the corresponding core-shell imprinted particles. The approach will open a new window of interest to the exploration of hollow microspheres and provide new opportunities in the applications involved in the uptake of target species and the capsulation of drugs. In other work, Ki *et al.* [91] also prepared the molecularly imprinted nanocapsules without holes through a one-step microemulsion polymerization.

### 3.3. Molecularly Imprinted Nanowires, Nanotubes and Nanofibers

Since carbon nanotubes were discovered, various organic/inorganic nanowires/nanotubes have been synthesized via alumina templates, wet chemistry and vapor disposition in the past two decades, and are widely used as sensory materials of chemical/biological detections [92-96]. However, molecular imprinting on these one-dimensional nanostructures will endow the nanowires/nanotubes with molecular recognition functions, further expanding their application fields [14, 15, 65, 69]. Yan and co-workers [97, 98] first reported the creation of MIP filaments by a soft lithography microfabrication. The imprinting polymerization reaction took place within microchannels formed between a silicon wafer and a microfabricated poly(dimethyl siloxane) (PDMS) stamp. One main problem connected with PDMS microchannels is that the stamp material is not compatible with the most commonly imprinting systems containing acrylate, methacrylate, or styrene monomers. Recently, a porous alumina template was introduced into fabrication of imprinted nanowires/nanotubes by Yang *et al.* [65, 69]. The imprinting of proteins and amino acids were carried out through immobilizing template molecules on the inner wall of a porous alumina membrane. The imprinting polymerization selectively occurred within the nanochannels of porous alumina template. After the supporting alumina was removed by chemical dissolution, polymer nanowires/nanotubes with the imprinted recognition sites of biological molecules can be obtained.

**Figure 5.**
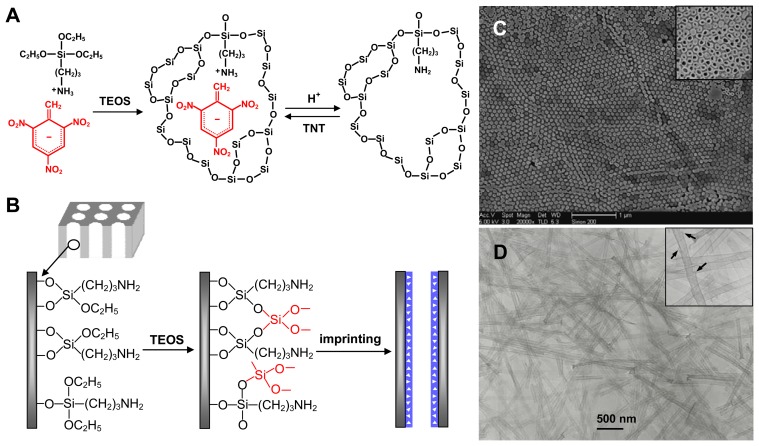
(A) Schematic illustration for the molecular imprinting mechanism of TNT in silica matrix through the anion-cation pair and gelation reaction. (B) The formation mechanism of TNT-imprinted silica nanotubes within APTS-modified porous alumina membrane. (C) Top-view SEM image of the TNT-imprinted silica nanotube array embedded inside alumina membrane after the membrane surface was mechanically polished. (D) TEM image of individual TNT-imprinted silica nanotubes liberated from the alumina pores by dissolving the alumina membranes. Insets are the high-magnification SEM and TEM images, respectively. Reprinted with permission from [15]. Copyright 2008 American Chemical Society.

In recent work, we demonstrated a surface molecular self-assembly strategy for the preparation of TNT-imprinted polymer/silica nanowire or nanotube arrays [14, 15]. As shown in Figure 5, a porous alumina membrane was first modified with 3-aminopropyltriethoxysilane (APTS), forming an APTS monolayer at the pore walls. TNT templates can spontaneously assemble onto the APTS monolayer by a strong charge-transfer complexing interaction. Simultaneously, the APTS monolayer can also drive the selective occurrence of imprinting polymerization along the pore walls. The TNT-imprinted polymer nanowires/nanotubes and silica nanotubes with ultrathin wall thickness of 15 nm were synthesized by controlling the reaction conditions. The capacities of binding TNT by the imprinted polymer nanowires and nanotubes are 2.5-3.0 fold that achieved with normal imprinted bulky particles. Moreover, the imprinted nanowires and nanotubes have nearly a 4 and 6 fold increase in the rate of TNT molecule binding, respectively. On the other hand, the imprinted silica nanotubes with ultrathin wall thickness exhibit greater capacity and faster kinetics of uptaking target species due to a better site accessibility and lower mass-transfer resistance. The high-quality imprinted nanowires/nanotubes are the ideal forms of materials for applications in chemo/biosensors and bioassays.

Chronakis *et al.* [99, 100] recently developed two simple electrospinning methods for the preparation of molecularly imprinted nanofibers. In one case, they showed that imprinted nanofibers can directly be produced using a simple electrospinning solution containing the polymerizing precursors and template molecules [99]. After removal of the templates such as the herbicide 2,4-dichlorophenoxyacetic acid (2, 4-D) by solvent extraction, the imprinted binding sites were left in the nanofibers. A more general approach is to use pre-prepared MIP nanoparticles as a starting material to produce composite nanofibers by electrospinning [100]. MIP nanoparticles can be easily encapsulated into nanofibers by the electrospinning, and the resultant composite nanofibers are very stable and maintain favorable molecular recognition capabilities.

### 3.4. Molecularly Imprinted Nanofilms

Of the various imprinted nanomaterials, nanofilms with a thickness smaller than one micrometer are one of the most desirable forms of the feasible applications in chemosensors, and have thus attracted intense research interest in recent years [101-107]. In addition to large surface-to-volume ratio and fast binding kinetics, the most remarkable advantage is that the molecularly imprinted nanofilms can be synthesized directly on the surfaces of electrochemical electrodes [40], quartz crystal microbalances (QCM) [101, 102] and surface plasmon resonators (SPR) [103] for the detection of target analytes. The simplest approach to MIP nanofilms is to spin-coat the prepolymerizing precursors of imprinting materials onto a flat substrate, followed by a cure process at higher temperature [71, 101, 104]. The film thickness can easily be controlled by changing the speed of spin-coating, and the porosity of film can also be adjusted by the addition of other low molecular-weight polymers. For example, a hexachlorobenzene-imprinted polymer film with a thickness of 400 nm was obtained by polymerizing a spin-coated layer of the monomers at the surface of a QCM chip [104]. The sensors demonstrated both high selectivity and sensitivity with a detection limit of down to 10^-12^ M, while exhibiting an exceptionally fast response time of ∼10 s. Furthermore, Schmidt *et al.* [71] used different linear polymers to adjust the morphology of spin-coated MIP nanofilms. Removal of the linear polymer by solvent extraction resulted in different porous structures in the propranolol-imprinted nanofilm.

Recently, a surface sol-gel process was used to prepare molecularly imprinted TiO_2_ nanofilms by hydrolysis of titanium alkoxide in the presence of organic carboxylic acids [105]. The ultrathin TiO_2_ layer with 10∼20 nm thickness exhibited a complete rebinding during the period of 40-60 s, and the rebinding capacity was 11-16 fold that of the corresponding nonimprinted nanofilms. More recently, Tatemichi *et al.* [70] prepared the pepsin-imprinted organic/inorganic hybrid nanofilms for the protein recognition by the combination of liquid-phase TiO_2_ deposition with molecular imprinting. The imprinted nanofilms exhibited a high molecular selectivity to the imprinted target species. The binding constant of pepsin on the imprinted nanofilms was 7.3×10^5^ M^-1^, which was 10 times higher than that of albumin. In addition, Sreenivasan [106] developed a post-coating strategy for the imprinting of chiral specific sites at the surface of polyurethane film with a thickness of 150 nm. The selectivity factor was high, up to 24.24.

The layer-by-layer (LbL) assembly of opposite-charged polyelectrolytes had been widely used to synthesize various functional nanofilms and nanocapsules. Recently, Shi *et al.* [81] developed a novel approach for the generation of stable molecularly imprinted sites in polymeric multilayer nanofilms on a flat substrate by LbL assembly of photosensitive polyelectrolytes, followed by the photochemical crosslinking of the multilayers. The thickness of LbL films is tunable at the nanometer scale by controlling the number of layers. The imprinted multilayer film exhibited a reproducible and rapid molecular loading and unloading ability. The loading process reached a saturation value at about 100 s and the binding constant of template molecule to the imprinted site was estimated to be 2×10^5^ M^-1^. On the other hand, the LbL graft polymerization technology was also developed to imprint different molecules at the multilayer nanofilms [72].

In addition, the nanostructured molecularly imprinted films that consist of numerous nanosized building blocks can also be made by the employments of nanofabrication technologies for direct application in selective separations [79, 107]. For example, Yang *et al.* [107] synthesized a silica nanotube film by the use of nanoporous alumina membranes through a sol-gel procedure. The molecularly imprinted films were directly used to selectively separate the naturally occurring estrogen. Meanwhile, with the use of nanomolding, Vandevelde *et al.* [79] further prepared propranolol-imprinted film that is built up of upright nanofilaments with 200 nm in diameter and 2 μm in length. The MIP nanostructured films can not only increase surface area and accessibility of the binding sites, but also allow for the tunability of surface properties such as wetting property.

## 4. MIP-Based Chemosensors

MIPs have been increasingly used for the development of chemo/biosensors [42-46], because in practical applications as synthetic recognition elements they have many advantages, such as high stability, low cost and ease of preparation. The key to the biomimetic sensors is to establish a reliable link between the target binding event and transducer. Therefore, a major concern for the development of MIP-based sensors is how to measure the analyte binding at MIP materials. Typically, the MIP-based sensors are fabricated by assembling MIP materials onto the surface of transducer, and thus the analyte binding is transformed into a measurable signal (Figure 1B). In general, the efficiency of sensors doesn't only depend on the selectivity and sensitivity of MIPs to target species, but also on the approaches of signal output. The optimal transduction approach to a readable signal output can be expected to maximize the selectivity and sensitivity of sensors. In principle, many physical measurements such as electrochemical voltammetry, fluorescence, piezoelectricity and surface plasma resonance can be used for the signal detection in MIP-based sensors [42-46]. One should consider the properties of target analytes and the forms of MIPs to determine what transducer is used. For example, electrochemical voltammetry is an excellent approach for measuring the binding of electroactive analytes at the MIP nanofilms [108]. During the past ten years, the literatures on the development of MIP-based sensors, in particularly electrochemical [108-116] and optical [117-135] sensors, have been dramatically growing. At the same time, mass sensitive transducers are increasingly popular since the approach has the advantage, theoretically at least, that it can be universally applied to a broad range of targets [136-147]. With insight into the different types of sensing, we will summarize the recent advances on electrochemical and optical sensors and mass sensitive devices.

### 4.1. Electrochemical Sensors

MIP-based electrochemical sensors were first reported in the early 1990s by Mosbach's group [108]. They described the integration of a phenylalanine anilide imprinted polymer into a field-effect capacitance sensor and reported a significant reduction in the overall capacitance when the sensor was exposed to the target species. The capacitance sensors based on MIPs were also fabricated and used to detect many other analytes such as amino acid derivatives with a detection limit of 500 ppm [109], and barbituric acid with a detection limit of 3.5 ppm [110]. During the past decade, remarkable progress in MIP-based electrochemical sensors have been achieved by the use of conductometric/potentiometric measurements and MIP nanomaterials, greatly extending the range of detected targets and improving the sensitivity, selectivity and simplicity of electrochemical sensors [42, 45, 109-116]. The electrochemical sensors are most commonly fabricated by installing MIP nanomaterials, as recognition elements, onto the surface of electrode. The changes of current and peak voltage at cyclic voltammetry upon the analyte binding can sensitively respond to the concentration and kind of analytes, respectively, because of the oxidation or reduction of analytes at the MIP-modified electrode. Recently, Kan *et al.* [111] reported a novel electrochemical sensor to detect the neurotransmitter dopamine by modifying the glassy carbon electrode with a composite of multiwalled carbon nanotubes (MWNTs) and dopamine-imprinted polymers. The MWNT-MIP-modified electrode did not only possess a rapid dynamic binding with an equilibrium period of 30 min, but also exhibited a high selectivity and sensitivity toward dopamine with a linear range of 5.0×10^-7^ to 2.0×10^-4^ M.

**Figure 6.**
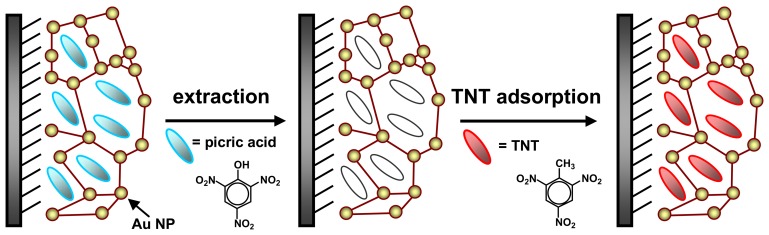
Imprint of molecular recognition sites for TNT in an oligoaniline-crosslinked Au nanoparticle (NP) film polymerized at the Au electrode. Reprinted with permission from [112]. Copyright 2008 American Chemical Society

Recently, Riskin *et al.* [40] demonstrated that polyphenol can be electropolymerized on a Au electrode to fabricate the MIP-modified electrode for the detection of herbicide molecules, *N*,*N*′-dimethyl-4,4′-bipyridinium (MV^2+^). The template-phenol interactions provided a driving force for the formation of MV^2+^-imprinted sites in the electropolymerized polyphenol film. When the MIP-modified electrode was used to detect the herbicide in solution, the currents rapidly leveled off to an equilibrium value after 20 min, and there was 14-fold increase in the binding affinity of MV^2+^, as compared to the non-imprinted film. Meanwhile, the current response at the MIP electrode upon the structurally-analogous 1,2-bis-4,4′-methylbipyridinium ethylene was 8 fold lower than that observed with MV^2+^. Just recently, they [112] reported another electrochemical sensor for the detection of TNT with enhanced sensitivities by imprinting of structure-like picric acid as substituting templates in the composite film of Au nanoparticles (Au NPs) and conductive polymers (Figure 6). The imprinting of molecular recognition sites into the *π*-donor oligoaniline-crosslinked Au NPs structure greatly enhanced the detection sensitivity. As little as 46 ppt of TNT (200 pM) can be clearly detected by the modified electrode, which was 370-fold the sensitivity of the *p*-aminothiophenolate monolayer-modified electrode. In contrast to other sensor systems, the imprinted *π*-donor Au nanoparticle crosslinked array is a highly sensitive electrochemical approach for the ultrasensitive detection of TNT.

Meanwhile, Levon and coworkers [113-115] had developed various interesting potentiometric sensors based on the surface imprinting technique coupled with a nanoscale transducer, indium tin oxide (ITO). An octadecylsiloxane monolayer was covalently bound to ITO-coated glass surface in the presence of templates. After extraction of templates, potentiometric measurements showed selective detections of the imprinted target species, such as methyl phosphonic acid with a detection range of 5.0×10^-5^ to 0.62 M [113], dipicolinic acid with a detection range of 1.5×10^-6^ to 0.0194 M [114], and *N*-carbobenzoxyaspartic acid with a detection range of 5.0×10^-6^ to 1.2×10^-2^ M [115]. Moreover, the response times were surprisingly short due to the imprinted ultrathin monolayer, i.e. ∼a few seconds except for the last one which is ∼2 min.

### 4.2. Optical Sensors

Of various signal transducers, optically addressable sensors based on fluorescent “turn-on” or “turn-off” mechanism have been demonstrated to be highly desirable for a variety of small molecular analytes in many challenging environments, due to their high signal output and feasible measurements [43-46]. However, the fluorescent sensors with a high sensitivity have also faced a challenge of selectivity. Therefore, great efforts have recently been made to prepare the fluorescent sensors using molecular imprinting methods, and many successful examples have been reported on the fluorescent detections of some analytes [117-129]. Most of the strategies involve in the design and use of fluorescent ligands and fluorotag-ligand conjugates in the preparation of the fluorescent sensors. The fluorescent functional monomers are coupled with imprinted sites, exhibiting fluorescence enhancement or quenching upon the analyte binding.

**Figure 7.**
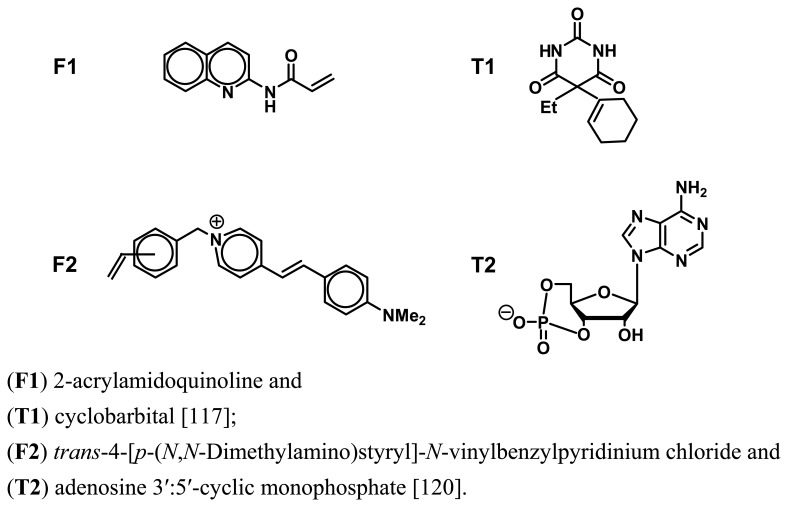
Fluorescent functional monomers (**F**) and their corresponding template molecules (**T**):

For example, Takeuchi and co-workers [117] designed and synthesized 2-acrylamidoquinoline (Figure 7, **F1**) as a fluorescent functional monomer with a polymerizable acrylate moiety and a fluorescent hydrogen-bonding moiety. The template cyclobarbital (**T1**) was imprinted into a polymer matrix by using the fluorescent functional monomer, in which the remarkable fluorescent enhancement upon the hydrogen bonding of the target **T1** into the imprinted sites was observed. The fluorescent sensor demonstrated the ability to signal the presence and concentration of the analyte with a detection range of 0.1-2.0 mM. Almost no or only a small fluorescence enhancement was observed on exposure to other structurally related compounds. Similarly, recent research has also demonstrated that the formation of the boronic ester bond between the boronic ligand and *cis* diols at the fructose-imprinted sites could lead to the significant fluorescence enhancement [118, 119]. The MIP-based fluorescent sensor can provide a sensitive detection for D-fructose by the fluorescent enhancement of 80 % for 100 mM D-fructose.

**Figure 8.**
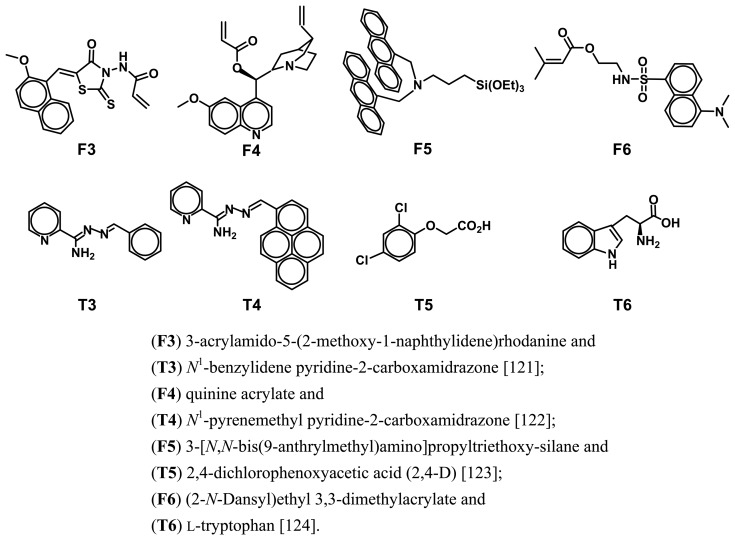
Fluorescent monomers (**F**) and the corresponding target analytes (**T**):

On the other hand, Turkewitsch *et al.* [120] reported the first example to use a fluorescent monomer **F2** (Figure 7) to prepare fluorescence-quenching sensor for the detection of cAMP (**T2**). The incorporation of the fluorescence probe into the pre-polymerization system resulted in the MIPs that can produce a quenching response to the rebinding of target **T2**. The MIP-based fluorescent sensor displayed a 20 % fluorescence quenching in the presence of 1 mM cAMP, whereas almost no effect was observed for the similar structural molecules. A main drawback of the imprinted fluorescent polymer is its high background signal. Another alternative fluorescent system was described by Rathbone and Ge [121, 122]. They synthesized a novel fluorescent monomer (Figure 8, **F3**) that were crosslinked into MIPs for the fluorescent-quenching detection of **T3** [121]. Dramatic fluorescence quenching approaching background levels was observed when the fluorescent sensor was exposed to its target species. The rebinding of template led to a 24.4-fold reduction in fluorescence intensity while the similar analytes gave a mere 1.7-fold quenching. The fluorescent quenching approach has recently further been validated by membrane-bound fluorescent imprinted polymer through using quinine acrylate (**F4**) to imprint the molecule **T4** [122].

In addition to the abovementioned fluorescence enhancement, photoinduced electron transfer [123], quencher-analyte competition adsorption [124] and chemiluminescene [125, 126] have been extensively explored to signal the analyte binding events. Photoinduced electron transfer has been a very popular mode of sensing in fluorescent molecular recognition in recent years [123, 127, 128]. It was demonstrated that the use of electron transfer mechanism as a means of signal transduction is feasible for the fluorescent detection of non-fluorescent analyte. Leung *et al.* [123] reported a sol-gel molecularly imprinted luminescent sensor fabricated by using a tailor-made organosilane (Figure 8, **F5**) as fluorescent functional monomer and 2,4-D (**T5**) as template molecule. Luminescence of **F5** was greatly enhanced by the formation of acid-base ion pairs with 2,4-D, because of the suppression of photoinduced electron transfer quenching on the anthryl fluorophore emission. Therefore, the imprinted sol-gel materials exhibited a selective fluorescent response to 2,4-D by the significant enhancement of fluorescence. A gradually rising trend in luminescent intensity was observed with increasing 2,4-D concentration from 10 to 166.6 *μg* mL^-1^, while the control materials showed negligible response in luminescent intensity.

Recently, Liao *et al.* [124] reported a general approach to develop a fluorescence sensor for the detection of non-fluorescent analytes by quencher-analyte competition adsorption. A fluorescent monomer (**F6**) was incorporated into **T6**-imprinted polymer. The fluorescence of this polymer could be quenched by an external quencher 4-nitrobenzaldehyde. With addition of target **T6**, the fluorescence intensity of the imprinted polymer gradually recovered in a concentration-dependent fashion through the displacement of the quencher from the binding sites by **T6**, allowing the binding events to be detected easily. The detection limit was down to single digit μM concentration.

Another consideration is to prepare the chemiluminescent (CL) flow-through sensors based on the combination of molecular imprinting and CL. When a suitable CL system was chosen, the MIP-CL sensors have usually a high sensitivity and selectivity. Lin and Yamada [125] prepared the first MIP-CL sensor for the detection of dansyl-L-phenylalanine. The polymer particles with the imprinted sites of dansyl-L-phenylalanine were used as a recognition element in CL flow-through sensor. When the HSO_5_^-^/Co^2+^ solution flowed through the polymer particles, the target analytes adsorbed on the MIP particles were oxidized to decompose and gave out CL emission with a low detection limit of 4×10^-7^ M. To date, there are only a few reports on MIP-CL sensors for limited analytes, and it may be due to the lack of suitable CL systems [125, 126].

Small organic luminophores, however, tend to bleach or degrade over time or in the presence of light and heat. Recently, fluorescent conjugated polymers with high quantum yield and photostability have found wide applications as a foundation for building fluorescent sensors [129-131]. The conjugated polymer-based sensors as compared to small molecule sensors can provide an amplified response to an analyte binding event by intrinsic exciton migration from large area to the sites where analyte binding occurs. Nesterov and co-workers [129] recently prepared the microparticles of molecularly imprinted fluorescent conjugated polymer (MICP) for the selective detection of nitroaromatic compounds. Exposure of MICP to TNT vapor for 10 min caused a substantial decrease in fluorescence intensity, and the fluorescence can remarkably recover after keeping them for 10 min in air, and completely recover in 1 h.

**Figure 9.**
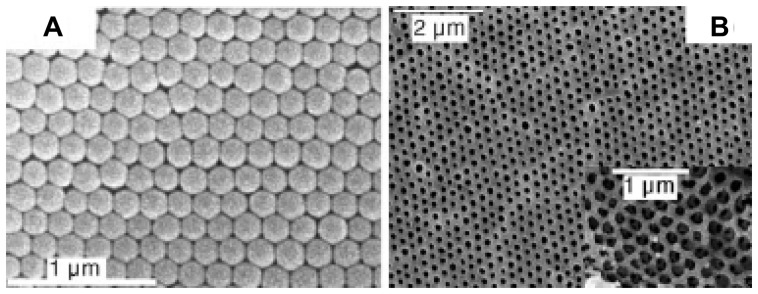
(A) SEM image of colloidal crystal formed with SiO_2_ spheres of diameter 186 nm. (B) Top-view SEM image of the fabricated photonic MIP film. Inset: Magnified cross-section SEM image of MIP film showing the interconnection between the macropores of the fabricated MIP film. Reprinted with permission from [134]. Copyright 2006 Wiley-VCH Verlag GmbH & Co. KGaA.

The stability and response reversibility significantly increase the value of MICP for potential chemosensing applications in fluorescent sensors. On the other hand, inorganic luminophores such as semiconducting nanocrystals and quantum dots are also very attractive as fluorescent labels in MIP-based sensors [132, 133]. When quantum dots are located close to the imprinted recognition sites, the analyte binding at the recognition sites can quench the photoluminescence of quantum dots because of the Förster resonance energy transfer [133]. The emission intensity exhibited a 4∼5-fold reduction in the presence of 200 ppm analytes (such as caffeine, uric acid, L-cysteine and estriol), while the non-imprinted composite did not show any change in photoluminescence emission. The uses of fluorescence labeling and chemiluminescence in MIP-based optical sensors usually involves in the complex chemical procedures. Recently, a novel “self-reporting” MIP-based optical sensor has been developed through combining molecular imprinting into photonic crystals. Hu *et al.* [134] adopted colloidal silica nanoparticles as template to prepare highly ordered 3D macroporous hydrogel films with molecularly imprinted sites (Figure 9). The films directly generated a readable optical signal (self-reporting) upon binding a target analyte. The optical absorption band of the macroporous MIP film shifted gradually to shorter wavelength with the increase of concentration of L-dopa. In contrast, the absorption band remained almost unshifted for the MIP films exposed to various concentrations of D-dopa. The current method emphasizes the use of a readable optical signal caused by structural changes upon analyte binding, and this special MIP film has many advantages such as quick response time and high sensitivity and selectivity. Another non-labeling optical sensor was just recently developed for the detection of explosive TNT by utilizing integrated optical waveguide attenuated total reflection spectrometry by depositing the submicron thick films of molecularly imprinted sol-gel polymers onto the waveguide surface as the sensing layer [135]. The limit-of-detection to TNT vapor was five parts-per-billion (ppb) in ambient air at a flow rate of 40 mL min^-1^. The sensor is highly specific for TNT due to the selective binding at recognition sites and the subsequent generation of the TNT anions.

### 4.3. Mass Sensitive Devices

In principle, the measurement of mass is the most general method suitable for the detection of any analyte since the mass is a universal property of matter. Piezoelectric devices such as a quartz crystal microbalance (QCM) can provide an extremely sensitive measurement to the mass of the analyte binding at the surface of piezoelectric materials through an accompanying decrease of the oscillation frequency of a piezoelectric crystal. The variation of vibrational frequency (Δ*F*) can be evaluated from Sauerbrey equation [147]: $\Delta F=-2.3\times 10^{6}\times F_{0}^{2}\times (\text{M}/\text{A})$, where *F*_0_ is a fundamental frequency of piezoelectric crystal, *M* is the mass of absorbed species, and *A* is the adsorbing surface area. The theoretical detection limit if using quartz crystals is about 10^-12^ g. Although the volume of literature on MIP-based piezoelectric sensors has increased at a relatively slow rate compared with electrochemical and optical sensors, the synergetic advantages of the selectivity provided by MIP with the sensitivity provided by piezoelectric sensing makes the sensors almost universally applicable with good limits of detection, low cost and the possibility of easy miniaturization and automation [45, 136-147]. A recent review has summarized the applications of the piezoelectric sensors in the selective detections of small molecules [136]. Here we mainly review the recently released publications on applications of MIP nanomaterials in piezoelectric sensors and summarize the extension of their applications from small molecules to biomacromolecules and to bulky analytes such as microorganisms and cells.

**Figure 10.**
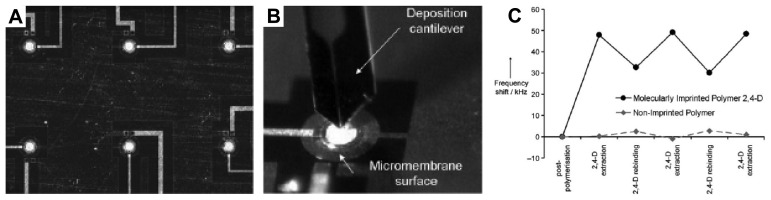
Image of (A) a matrix of piezoelectric micromembranes with a global radius of 100 µm and (B) a cantilever loaded with MIP precursor solution during deposition onto a micromembrane. (C) Reproducibility of resonance-frequency measurements for successive 2,4-D removal and rebinding cycles. Reprinted with permission from [146]. Copyright 2007 Wiley-VCH Verlag GmbH & Co. KGaA.

A MIP-based QCM sensor is usually fabricated by immobilizing a MIP layer onto the surface of QCM as a recognition element. Accordingly, the sensing system can selectively detect the specific binding of target analyte at the MIP materials [136-142]. Krozer [137] reported the QCM sensor with dissipation (QCM-D) by coating the sensor surface with pre-made molecularly imprinted nanoparticles. The nanoparticles were physically entrapped into a thin poly(ethylene terephthalate) (PET) layer spin-coated on the transducer surface. By controlling the deposition conditions, a high nanoparticle loading can be gained in the stable PET layer, allowing the recognition sites in nanoparticles to be easily accessed by the test analytes. The highest uptake of the nanoparticle film to propranolol corresponded to approximately 2 nmol cm^-2^ or about 1x10^15^ molecules cm^-2^. The detection limit of the MIP-QCM sensor was about 10 μM, and the chiral recognition and discrimination between *R*- and *S*-propranolol can also be achieved. Similarly, Chan [138] described the use of the thin permeable films of MIPs as biomimetic recognition materials on QCM surface. The sensor can provide a high enantioselectivity and sensitivity for the discrimination of L- and D-tryptophan enantiomers with a detection limit of 8.8 μM. On the other hand, the direct surface polymerization on a self-assembled alkanethiol monolayer of a gold-coated QCM resonator can also produce the thin MIP coating layer to build a MIP-QCM sensors [139, 140]. For example, Piacham *et al.* [140] studied the preparation of thin MIP nanofilms on the surface of QCM. The thickness of the MIP film was below 50 nm. The selective recognition of target analytes can be easily detected by underlying quartz crystal resonator. When used in a flow injection analysis system, the assembled QCM sensor generated a large frequency change (>30 Hz) upon encountering a small amount of analyte (0.19 mM), and had a very short response time (<1 min), and displayed certain chiral selectivity towards the original template, S-propranolol, at a concentration higher than 0.38 mM. Haupt *et al.* [141] reported another enantioselective QCM-MIP sensor. Using *S*-propranolol as a template, a poly(trimethylolpropane trimethacrylate-co-methacrylic acid) membrane was prepared by sandwiching the pre-polymerization solution between a gold-coated QCM chip and the surface of a UV lamp. The resultant sensor showed good enantioselectivity for the ‘*S*’ enantiomer when compared to the ‘*R*’ enantiomer. In addition to imprinted polymer films, inorganic composite nanofilms have recently been prepared and used in the QCM sensors. Yang *et al.* [142] fabricated the glucose-sensitive TiO_2_ ultrathin composite films by molecular imprinting on a gold electrode of QCM for the detection of monosaccharides. The largest binding capacity was 2.3 fold that of the corresponding non-imprinted films. Since silicon microfabrication technology allows the production of sensors with multiplexing capabilities and high integration, microelectromechanical system (MEMS) as transducers coupled to sensitive layer have greater potential in ultrasensitive piezoelectric sensors [143-145]. Recently, Haupt [146] reported the first experimental proof of concept of the combination of resonant MEMS with MIPs to fabricate the resonant piezoelectric micromembrane sensors. The micromembrane arrays were individually coated with MIPs by using a cantilever-based deposition tool to upload the molecular imprinting precursor solution, as shown in Figure 10A and B. The droplets were immediately polymerized under UV light to form a sensitive layer of MIPs. With the herbicide 2,4-D, a reproducible frequency shift of -16.5 kHz was observed at the 10 μM concentration, while no variation was measured on the non-imprinted micromembrane (Figure 10C). The miniaturized instrumentation offers new opportunities for the portable biosensors dedicated to environmental analysis.

The determination of proteins is very important in quick medical diagnostics and modern drug discovery [48, 49]. The traditional detection approaches such as dye labeling and electrophoresis usually suffer from poor selectivity and sensitivity when the specimen is a protein mixture. The MIP film coupled with QCM may be a potential analytical method for the detection of proteins. In the case of protein imprinting, MIP film is a most desirable form of materials since the removal of high-molecular protein template becomes much easier than in normal bulky MIP monoliths [48, 49, 62, 147]. Chou [147] recently prepared the albumin MIP film to analyze albumin protein mixture by MIP-QCM sensor. The albumin MIP film was successfully coated on the QCM gold electrodes with amino, hydroxyl and carboxyl ligands by ultraviolet light-inducing polymerization. With respect to adsorption selectivity, the MIP-QCM sensor exhibited a high response to albumin than other serum proteins, in which adsorption mass ratio of cytochrome *c*:lysozyme:albumin:myoglobin was 160:1:1942:30. In addition, the MIP-QCM results also showed a good match with the data of the clinical assay in the test of human serum.

Furthermore, the rapid and inexpensive screening of viral and bacterial infections is urgently needed for preventing the spread of infection in animal and plant populations [148-151]. However, the detections of viruses and bacteria are generally time-consuming and expensive tasks because the biological detection needs a complex process including incubation, separation, dying and microscopy [148-151]. The bioimprinted QCM sensors may provide a fast and selective biodetection by the combination of bioimprinting and QCM. Hayden recently developed the surface imprinting techniques on polymer-coated QCM to detect tobacco mosaic viruses (TMV) [152, 153] and living yeasts [154] in aqueous media. The imprinting cavities or trenches on the polymer surface mimicking the shape and surface functionality of the viruses and bacteria served as recognition sites for their rebinding. The sensors were applicable to TMV detection ranging from 100 ng mL^-1^ to 1 mg mL^-1^ [153], and allowed a selective on-line monitoring of the yeast cell concentrations in water over 5 orders of magnitude [154]. Moreover, they continued to expend the bioimprinting concept to the recognition of mammalian cells, which is a greater challenge because of their lower mechanical stability compared to microorganisms [63, 154, 155]. Erythrocyte-specific interactions with recognition sites on surface imprinted polyurethane were applicable for blood-group typing of the main ABO antigens [63]. The interesting finding in MIP-QCM sensor was highly relevant for clinical applications in serology.

## 5. Conclusions and Future Perspectives

These research described above has clearly shown that various nanotechnologies are increasingly being adopted in the preparation of MIP materials and the fabrication of MIP-based sensors. MIP nanomaterials with different forms such as nanoparticles, core-shell/hollow nanoparticles, nanowires, nanotubes and nanofilms have been synthesized in a controlled way by the employments of nanotechnologies and surface chemistry. The imprinting of molecular recognition sites at nanostructures has greatly improved the removal of templates and the binding capacities and kinetics of molecular recognition, compared with the traditional imprinted bulky materials. Nanosized MIP materials with high surface-to-volume ratio are characterized by their capability of binding target molecules with similar affinity and selectivity to those of antibody, whereas they offer a higher physical/chemical stability and better engineering possibility than biological receptors. In particular, MIP nanomaterials as molecular recognition elements have exhibited remarkable advantages for the applications in biomimetic chemo/biosensors. The ultrasensitive detections of some analytes have also been achieved by the combination of MIP nanomaterials with transducers. Up to date, although many successful examples for the development of MIP nanomaterials and their applications in chemo/biosensors were reported, several key problems associated with the development of MIP nanomaterials and sensing systems need to be solved before commercial applications start. The main challenges include: (1) the enhancement of specific molecular affinity and reduction of non-specific adsorption by the design of MIP nanostructure; (2) the development of a general synthesis protocol for MIP nanomaterials with uniform shape and size; (3) the engineering combination of MIP nanoarrays with transducer in a suitable format; (4) the development of multisensors with multiplexing capabilities and high integration through the use of nanofabrication. The future efforts to address these concerns by multidisciplinary approaches will progressively extend the real applications of MIP nanomaterials in chemo/biosensors as well as many other fields.
